# Clinical and Experimental Factors Influencing the Efficacy of Neurofeedback in ADHD: A Meta-Analysis

**DOI:** 10.3389/fpsyt.2019.00035

**Published:** 2019-02-18

**Authors:** Aurore Bussalb, Marco Congedo, Quentin Barthélemy, David Ojeda, Eric Acquaviva, Richard Delorme, Louis Mayaud

**Affiliations:** ^1^Mensia Technologies SA, Paris, France; ^2^Child and Adolescent Psychiatry Department, Robert Debré Hospital, Paris, France; ^3^GIPSA-Lab, Université Grenoble Alpes, CNRS, Grenoble-INP, Grenoble, France

**Keywords:** ADHD, neurofeedback, meta-analysis, analysis of bias, EEG

## Abstract

Meta-analyses have been extensively used to evaluate the efficacy of neurofeedback (NFB) treatment for Attention Deficit/Hyperactivity Disorder (ADHD) in children and adolescents. However, each meta-analysis published in the past decade has contradicted the methods and results from the previous one, thus making it difficult to determine a consensus of opinion on the effectiveness of NFB. This works brings continuity to the field by extending and discussing the last and much controversial meta-analysis by Cortese et al. (1). The extension comprises an update of that work including the latest control trials, which have since been published and, most importantly, offers a novel methodology. Specifically, NFB literature is characterized by a high technical and methodological heterogeneity, which partly explains the current lack of consensus on the efficacy of NFB. This work takes advantage of this by performing a Systematic Analysis of Biases (SAOB) in studies included in the previous meta-analysis. Our extended meta-analysis (k = 16 studies) confirmed the previously obtained results of effect sizes in favor of NFB efficacy as being significant when clinical scales of ADHD are rated by parents (non-blind, *p*-value = 0.0014), but not when they are rated by teachers (probably blind, *p*-value = 0.27). The effect size is significant according to both raters for the subset of studies meeting the definition of “standard NFB protocols” (parents' *p*-value = 0.0054; teachers' *p*-value = 0.043, k = 4). Following this, the SAOB performed on k = 33 trials identified three main factors that have an impact on NFB efficacy: first, a more intensive treatment, but not treatment duration, is associated with higher efficacy; second, teachers report a lower improvement compared to parents; third, using high-quality EEG equipment improves the effectiveness of the NFB treatment. The identification of biases relating to an appropriate technical implementation of NFB certainly supports the efficacy of NFB as an intervention. The data presented also suggest that the *probably blind* assessment of teachers may not be considered a good proxy for blind assessments, therefore stressing the need for studies with placebo-controlled intervention as well as carefully reported neuromarker changes in relation to clinical response.

## 1. Introduction

Attention Deficit/Hyperactivity Disorder (ADHD) is a common childhood psychiatric disorder characterized by impaired attention and/or hyperactivity/impulsivity. Symptoms may persist in adulthood with clinical significance, which makes ADHD a life-long problem for many patients (2). The prevalence of ADHD is around 5% in school-aged children, thus affecting an estimated 2.5 million children in Europe (3). ADHD negatively impacts children's well-being, with many suffering from low self-esteem (4) and underachievement in school (5). Parents are equally affected, since the child's behavior is frequently attributed to bad parenting (6). From a societal point of view, ADHD also has a high financial impact: a 2013 survey in Europe estimated costs related to ADHD between 9, 860 and 14, 483 Euros per patient/year (7).

The diagnosis of ADHD primarily relies on questionnaire-based clinical evaluation (3), which can be supported by objective assessment metrics of executive function such as the Test of Variables of Attention (TOVA) (8), the Continuous Performance Test (CPT) (9), and the Sustained Attention to Response Task (SART) (10). Objective markers of brain function using electroencephalogram (EEG), functional Magnetic Resonance Imaging (fMRI), or Positron Emission Tomography (PET) are not considered to be useful for improving diagnosis at the individual level, but can help in differentiating groups of patients (11). In particular, different phenotypes of ADHD patients present with an increase in the EEG theta wave power (4–8Hz) and/or a decrease of EEG beta wave power (12–32Hz) in frontal areas, or a decrease in the EEG Sensorimotor Rhythm (SMR) power (13–15Hz) in the central area (12–15). A device using EEG to help clinicians more accurately diagnosis ADHD was cleared by the Food and Drug Administration (FDA) (16).

Psychostimulants are the most common treatment currently in use, and have proven to be efficacious (17, 18). However, their long-term effectiveness and side effects are still debated and form an active area of research (19–23). Moreover, ADHD children under medication commonly suffer from mild side effects such as loss of appetite and sleep disturbance, although serious adverse events are rare (18, 24). These drawbacks make some parents and clinicians reluctant to opt for such treatment, instead turning to non-pharmaceutical alternatives such as dietary changes (25) and behavioral therapy, which have been proven to be less efficacious (26).

Neurofeedback (NFB) is another non-pharmaceutical and non-invasive approach aiming at the reduction of ADHD symptoms (27–29). Shortly after the discovery of the brain's electric activity by Berger (30) and Durup and Fessard (31) demonstrated it could be voluntarily modulated, leading to a series of findings on the self-regulation of brain activity. The first indication of the therapeutic potential of brain activity operant-conditioning came 40 years later when Sterman et al. (32) found that training the SMR activity reduces the incidence of epileptic crisis in kerosene-exposed cats. The technique, then known as NFB, rapidly became the subject of investigation in various fields of neuropsychiatry including, most notably, ADHD (33–36).

NFB is a self-paced brain neuromodulation technique that represents brain activity in real-time using auditory or visual modulations, on which learning paradigms, such as operant conditioning (37) or voluntary control, can be applied. To deliver this intervention, neurophysiological time series are analyzed online in order to drive feedback applications such as serious games (38). The signal of interest should represent the activity of a population of neurons involved in attentional networks, which is translated into visual or auditory cues. The sensory feedback constitutes the rewards mechanism, promoting learning using, for instance, operant conditioning protocols (39). Operant conditioning enables neural plasticity, thus supporting the child in the task repetition (40), which is believed to result in long-lasting neuronal reorganization (41).

Several NFB protocols have been proposed and investigated for decreasing the symptoms of ADHD:

- protocols based on neural oscillations, using frequency-band power training: enhancing SMR (42), reducing theta (29) or enhancing beta (43), or a composite protocol such as enhancing beta while suppressing theta, also known as the Theta Beta Ratio (TBR) protocol (33, 44);
- protocols based on Slow Cortical Potentials (SCPs) training consisting of the regulation of cortical excitation thresholds by focusing on activity generated by external cues (45, 46);
- protocols to enhance Event-Related Potentials (ERPs): in particular, the amplitude of the P300 ERP can be considered as a specific neurophysiological marker of selective attention (47).

Moreover, NFB protocols can be personalized: some studies did not use the usual definitions of EEG band ranges but determined them thanks to the individual Alpha Peak Frequency (iAPF) (48), giving individualized NFB protocols (49–51).

NFB efficacy on the core symptoms of ADHD (inattention, hyperactivity, and impulsivity) has been the subject of several meta-analytic studies (26, 52–55). To date, studies have not reached a consensus on the efficacy of NFB; while Arns et al. (54) and Micoulaud-Franchi et al. (55) claim results in favor of its efficacy, especially on the inattention component highlighted by Micoulaud-Franchi et al. (55), other authors, such as Loo and Barkley (52), Lofthouse et al. (53), and Sonuga-Barke et al. (26) express their reservations, asking for further evidence from blind assessment.

The most recent meta-analysis addressing the efficacy of NFB was published by Cortese et al. (1), including a total of 13 Randomized Controlled Trials (RCTs). The results of this analysis are mixed: when based on parent assessments, which are not blind to treatment, they are significantly in favor of NFB, whereas when the evolution of symptoms is rated by teachers (considered as probably blind), the results are no longer significant. The authors concluded that further evidence from blind assessments is needed in order to support NFB as a treatment for ADHD symptoms. However, some of the choices made in this meta-analysis, which may have had an impact on the results, have since been debated by the community. Specifically, Micoulaud-Franchi et al. (56) criticized the use of an uncommon behavioral scale provided by Steiner et al. (57) for the teachers' assessments and the inclusion of a pilot study carried out by Arnold et al. (58).

As a result of these criticisms and the concurrent publication of new RCTs meeting Cortese et al.'s (1) inclusion criteria, we decided to update this meta-analysis and take the opportunity to investigate the impact of its controversial choices. While performing our investigation, we observed two shortcomings: the assumption that the difference between teacher and parent assessments can solely be explained by the placebo effect, and pooling together heterogeneous studies in terms of methodology and technical implementation. An interesting approach, albeit not commonly performed, to assess the NFB efficacy would be to analyze the specificity of the EEG changes with respect to trained neuromarkers (36). In our case, based on the data at our disposal, we used the technical and methodological heterogeneity of the NFB trials to our advantage rather than disadvantage by extending the previous work with a novel method, the Systematic Analysis of Biases (SAOB). Indeed, the NFB domain is characterized by clinical literature that is extremely heterogeneous: studies differ methodologically (for instance, random assignment and presence of a blind assessment), in the NFB implementation (for instance, number of sessions, session and treatment length, and type of protocol) as well as on the acquisition and processing of the EEG signal. Description and analysis of different types of NFB implementation was subject to several studies (59–62). However to the best of our knowledge, none of these studies has used statistical tools to quantify their influence on clinical endpoints.

Since methodological and technical implementations of studies are highly likely to influence their outcomes (63), we suggest identifying which of the factors independently influence the clinical efficacy with the use of appropriate statistical tools. In addition, we have made available all the raw RCT data we have used and a complete Python library for performing meta-analysis (64). Through doing so, we hope to foster the replication of our and previous studies and to facilitate similar future projects.

## 2. Materials and Methods

### 2.1. Inclusion Criteria

Search terms were directly taken from Cortese et al. (1) with the exception of the need for a control arm, which is detailed in Supplementary Material (65). The requirements included:

- studies have to assess NFB efficacy;
- subjects must have received a diagnosis of ADHD based on DSM-IV (66), DSM-5 (3), ICD-10 (67) criteria, or by a qualified psychiatrist;
- studies have to be written in English, German, Spanish, or French;
- studies have to include at least eight subjects in each group;
- patients must be younger than 25 years old;
- the publication has to disclose sufficient details about the data to compute required metrics for the ensuing analysis.

The studies satisfying all these criteria were included in the SAOB. In order to replicate and update Cortese et al.'s (1) meta-analysis, we applied the original inclusion criteria of their meta-analysis to our search (the main difference being the presence of a control group).

### 2.2. Outcome Definition

In the included studies, the severity of ADHD symptoms have been assessed by parents and, whenever available, by teachers. Cortese et al. (1) and Micoulaud-Franchi et al. (55) defined parents as Most Proximal (MPROX) raters who were not blind to the treatment, as opposed to teachers, who were considered as Probably Blind (PBLIND) raters. This distinction is intended to assess the amplitude of the placebo effect, where it is hypothesized that teachers, who are presumed to be more blind to the intervention, are less influenced in their assessment. Efficacy of NFB was measured using clinical scales, such as the ADHD-RS (68), on the following outcomes: inattention, hyperactivity/impulsivity, and total scores. The factor analysis was performed using the total score.

### 2.3. Meta-Analysis

The goal of a meta-analysis is to aggregate results from different clinical investigations and offer a consolidated body of evidence. To achieve this, it is necessary to assume some level of homogeneity in the design of the studies: inclusion criteria, and the presence and type of control (active, semi-active, or non-active). Because studies occasionally use slight variations of a clinical scale and because of the clinical heterogeneity of patients and control, the scores are standardized before being pooled into a Summary Effect (SE). The between-Effect Size (ES) is one such standardized metric, which we have implemented in this paper [see Supplementary Material (65)].

The meta-analysis was performed with a Python package developed for this work. The package offers a transparent approach for the choice of parameters in an effort to ease replicability. We have benchmarked it against RevMan version 5.1 [(69), UK, London] by replicating Cortese et al.'s (1) work. The code is made fully available on a GitHub repository (64), together with all the RCTs raw data we have used in the present study.

Before updating the Cortese et al. (1) work with recently published studies (70, 71), we decided to run a sensitivity analysis investigating the choices that later proved controversial (56). The investigated changes included:

- the between-ES of Arnold et al.'s (58) study was computed from the post-test clinical values taken after the completion of the 40 sessions, in contrast to Cortese et al.'s (1) report which used the results after only 12 sessions because the endpoint values were not available at the time of his study;
- the between-ES computed from the teachers' assessment reported by Steiner et al. (57) relied on the BOSS Classroom Observation (72). This is an atypical scale to quantify ADHD symptoms since the Conners Rating Scale Revised (73– 75), a well-defined (76, 77) and broadly used metric, was available in this study. Thus, we decided to compute the between-ES based on the Conners-3 already used in this study to compute the parents' between-ES.

As initially suggested by Cortese et al. (1) the analysis was run on two subgroups of studies with the two choices described above: one gathering studies following the standard protocol defined by Arns et al. (59) and a second including only participants not taking medications during the clinical trial.

### 2.4. Identify Factors Influencing the Neurofeedback

While revisiting the existing meta-analyses, it became apparent that the studies pooled together were highly heterogeneous in terms of methodological and practical implementation. For instance, all NFB interventions were pooled together regardless the quality of acquisition, the quality of EEG data, and the trained neuromarker. Equally, the methodological implementations varied significantly, requiring the “subgroup” analysis (for instance, gathering studies following standard protocols) that are somewhat arbitrary. To circumvent these limitations, we implemented a novel approach: the SAOB. With this method, the within-ES of each intervention was considered as a dependent variable to be explained by methodological and technical factors. Such analysis aims at identifying known methodological biases (e.g., blind assessments negatively associated with within-ES) and possible technical factors (e.g. a good control on real time data quality positively influences the treatment outcome).

#### 2.4.1. Identify and Pre-process Factors

We classified the factors influencing the efficacy of NFB into five categories: methodological, technical, demographics, and quality of the signal and acquisition. Factors were chosen based on that reported in the literature as presumed to influence ES, and categorized as follows:

- *the methodological biases*: the presence of a control group, the blindness of assessors, the randomization of subjects in controlled trials, and the approval of the study by an Institutional Review Board (IRB);
- *the population*: intake of psychostimulants during NFB treatment, the age range of children included, the severity of ADHD symptoms at baseline^1^, and the degree of engagement with NFB intervention;
- *the* *NFB* *implementation*: the protocol used (SCP, SMR, theta up, beta up in central or frontal areas, theta down), the presence of a transfer phase during NFB training, the possibility to train at home or at school with a transfer card, the type of thresholding for discrete reward, the number of NFB sessions, the length and frequency of the sessions, the length of the treatment, the individualization of the frequency bands based on the iAPF, and coupling NFB with Electromyogram (EMG)-Biofeedback;
- *the acquisition quality*: the presence of one or more active electrodes and the EEG data quality. The latter was coded as an indicator between 1 and 3, using the following criteria:
  *the type of electrodes used*: Silver Chloride (AgCl)/Gel or Gold (Au)/Gel;
  *the use of impedance mode*: a quality check of electrode contacts ensuring an inter-electrode impedance smaller than 40*kΩ*;
  *the level of hardware certificate*: compliance with ISO-60601-2-26 (78).
  A quality score equal to 3 was assigned if all the above criteria were satisfied. If at least one was satisfied the quality score was set to 2, otherwise the score was set to 1.
- *the signal quality*: online rejection (epoch rejected, feedback not computed) or correction (feedback computed on the denoised epoch) of Electro-Oculogram (EOG) artifacts, and online rejection of generic artifacts using an amplitude-based detection.

To prevent any bias in the analysis, the names of the factors were hidden during the entire analysis so that the data scientists (AB, QB, DO, and LM) were fully blind to them. The names were revealed only when the data analysis and results were accepted as valid: this included choice of variable normalization and validation of model hypothesis, as detailed below.

The pre-processing of factors for the analysis included the following steps: factors for which there were too many missing observations arbitrarily set to more than 20% of the total observations, were removed from the analysis. Furthermore, if a factor had more than 80% similar observations it was also removed. A study did not systematically correspond to an observation: when several clinical scales and/or raters were available in a study, each couple clinical scale–rater was considered as an observation. Categorical variables were coded as dummies, i.e., the presence of the factor was represented with 1 and its absence with 0. All variables were standardized by subtracting the mean and then dividing by the standard deviation (not applied before the decision tree described below).

#### 2.4.2. Explaining Within Effect Sizes With Factors

To compute the within-ES, the means of total ADHD scores given by parents and teachers were used. Moreover, in case studies providing results for more than one behavioral scale the within-ES scores were computed for each one as:

$$\begin{array}{l} \text{ES}=\frac{M_{\text{post,T}}-M_{\text{pre,T}}}{\sqrt{\frac{\sigma_{\text{pre,T}}^{2}+\sigma_{\text{post,T}}^{2}}{2}}}, \end{array}$$

where *M*_t,T_ is the mean of clinical scale, for treatment T, taken at time t (pre-test or post-test) and σ_t,T_ represents its standard deviation. With this definition, we focus on the effect of the treatment within a group (79) as commonly reported in the literature (36, 54, 70). This within-ES enables us to quantify the efficacy of NFB inside the treatment group. Finally, to avoid to break analysis methods assumptions, an outlier rejection was applied defining thresholds of acceptance as [μ − 3σ, μ + 3σ], with μ and σ being respectively the mean and the standard deviation of all within-ES computed (80).

The within-ES was then considered as a dependent variable to be explained by the factors (the independent variables). The following three methods, implemented with the Scikit-Learn Python (81, version 0.18.1) and the Statsmodels Python (82, version 0.8.0) libraries, were used to perform the regression:

- weighted multiple linear regression with Weighted Least Squares (WLS) (83);
- sparsity-regularized linear regression with Least Absolute Shrinkage and Selection Operator (LASSO) (84);
- decision tree regression (85).

The aim of the linear regression is to estimate the regression coefficients linking the factors to the within-ES. A significant coefficient (here and hereafter meaning significantly different from zero) indicates that the associated factor has an influence on NFB efficacy and its sign the direction of the effect. The WLS differs from a traditional linear regression estimated with Ordinary Least Squares (OLS) in that a weight is assigned to each observation in order to account for the multiplicity of reported clinical endpoints in some studies. In addition, the weight was also set as a function of the sample size to account for variations in sample sizes. Specifically, the weight of each study was taken as the ratio between the experimental group's sample size and the number of behavioral scales available. We also ran the analysis using the OLS method to assess the impact of the weights on the results.

The second linear method applied was the LASSO, which naturally incorporates variable selection into the linear model thanks to ℓ_1_-norm applied on the coefficients. A coefficient not set to zero means that the associated factor has an influence on NFB efficacy and its sign indicates the direction of the effect.

The last method used to determine factors influencing NFB was the decision tree (85), a hierarchical and non-linear method. This breaks down a dataset into smaller and smaller subsets using, at each iteration, a variable and a threshold chosen to optimize a simple Mean Square Error (MSE) criterion (86). A tree is composed of several nodes and leafs, the importance of which decreases from the top node, called the root node, downward.

Given that these methods are intrinsically different we compared their results. For instance, the decision tree captures variable interactions and can relate factors to within-ES in a non-linear fashion. On the other hand, the LASSO offers an elegant mathematical framework for variable selection. Further details are provided in the Supplementary Material (65).

## 3. Results

### 3.1. Selected Studies

**Figure 1:**
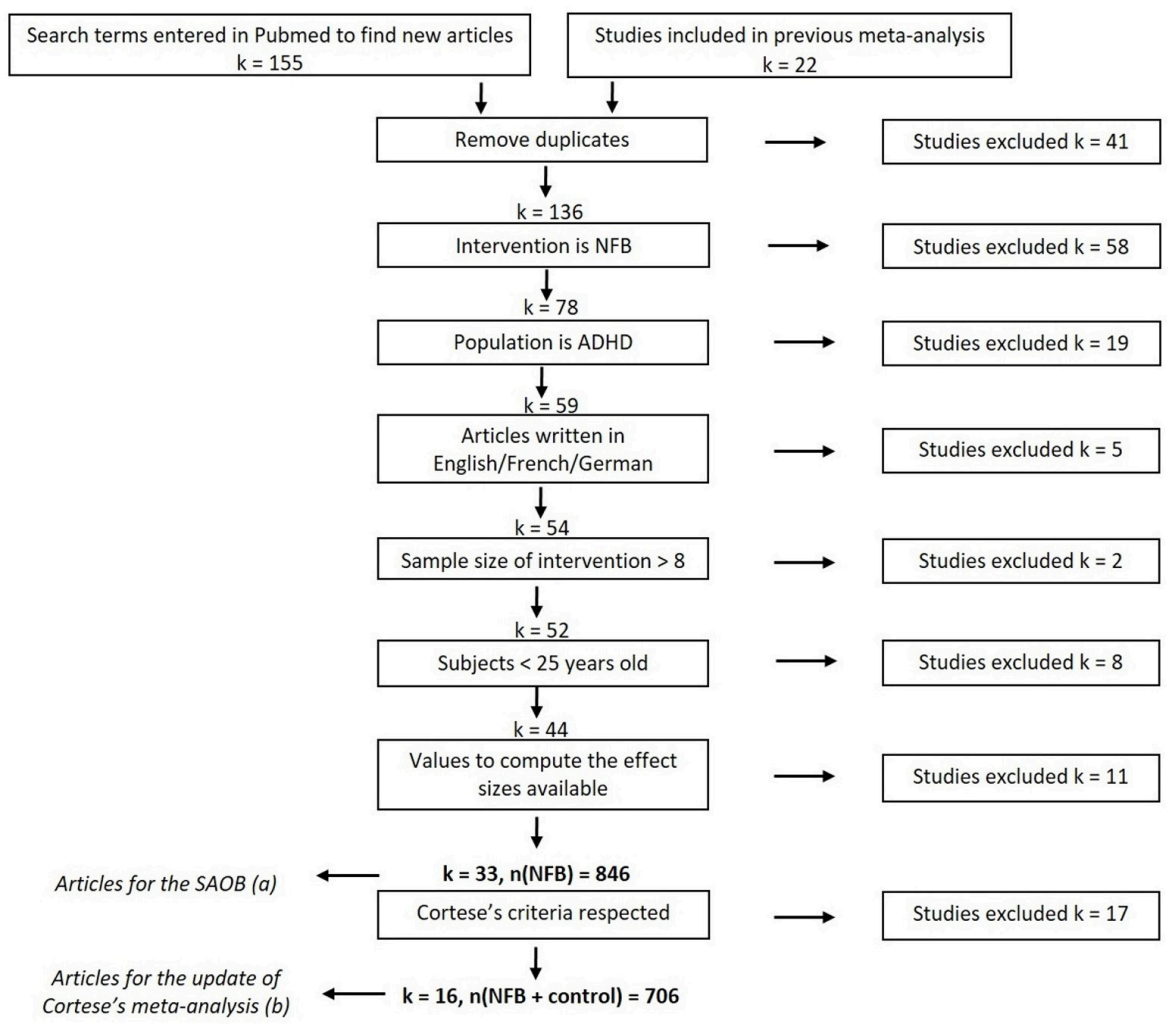
Flow diagram of selection of studies (last searched on February 12, 2018). The subset (a) corresponds to the Cortese et al.'s (1) inclusion criteria without the requirement of the presence of a control group. The subset (b) precisely corresponds to the studies included in Cortese et al. (1) and more recent works meeting the same criteria.

Search terms entered in Pubmed returned 155 results during the last check on February 12, 2018, including 22 articles used in previous meta-analyses on NFB. Following the selection process illustrated in Figure 1, 33 studies were included in the SAOB and 16 in the meta-analysis, as summarized in Table 1. The 33 studies selected for the SAOB followed Cortese et al.'s (1) criteria, with the exception of the requirement for a control group. Indeed, since within-ES were considered in this analysis, a control group was not required.

**Table 1:** List of all studies included in the three different analyses: (a) studies originally included in Cortese et al. (1) (last searched on August 30, 2015); (b) studies satisfying Cortese et al.'s (1) criteria (last searched on February 12, 2018); (c) studies satisfying Cortese et al.'s (1) criteria to the exception of the part relative to the control group (last searched on February 12, 2018).

**Analysis**			**Study**	**Year**	**Size of the NFB group**
			Arnold et al. (58)	2014	26
			Bakhshayesh et al. (87)	2011	18
			Beauregard and Levesque (42)	2006	15
			Bink et al. (88)	2014	45
			Christiansen et al. (74)	2014	14
			Gevensleben et al. (89)	2009	59
			Heinrich et al. (45)	2004	13
			Holtmann et al. (90)	2009	20
			Linden et al. (35)	1996	9
			Maurizio et al. (36)	2014	13
			Steiner et al. (91)	2011	9
			Steiner et al. (57)	2014	34
			van Dongen-Boomsma et al. (92)	2013	22
		a = Replicate Cortese et al. (1)	13 studies		297
			Baumeister et al. (71)	2016	8
			Bazanova et al. (51)	2018	17
			Strehl et al. (70)	2017	72
	b = Update Cortese et al. (1)		16 studies		394
			Bluschke et al. (75)	2016	19
			Deilami et al. (93)	2016	12
			Drechsler et al. (94)	2007	17
			Duric et al. (95)	2012	23
			Escolano et al. (50)	2014	20
			Fuchs et al. (96)	2003	22
			Geladé et al. (97)	2016	39
			Kropotov et al. (43)	2005	86
			Lee and Jung (98)	2017	18
			Leins et al. (99)	2007	19
			Li et al. (100)	2013	32
			Meisel et al. (101)	2014	12
			Mohagheghi et al. (102)	2017	30
			Mohammadi et al. (103)	2015	16
			Monastra et al. (104)	2002	51
			Ogrim and Hestad (105)	2013	13
			Strehl et al. (106)	2006	23
c = SAOB			33 studies		846

### 3.2. Meta-Analysis

The replication of Cortese et al.'s (1) results obtained are presented in Table 2:

- when computing the between-ES for Arnold et al. (58) with the values after 40 sessions of NFB, smaller between-ES were found as compared to those found by Cortese et al. (1), which was unexpected since the clinical efficacy is supposed to increase with the number of NFB sessions. These lower between-ES impacted the SE: they were slightly lower when computed with this choice although they nonetheless remained significant (see the three first lines of Table 2);
- when relying on the teachers' ratings from the Conners-3 to compute the between-ES of Steiner et al. (57), higher SEs were found in attention but not for total and hyperactivity score. However, this different choice of scale did not affect the statistical significance of the SEs (see the three last lines of Table 2).

**Table 2:** Comparison between Cortese et al. (1) results obtained with RevMan (69) and those obtained with the meta-analysis package with our choices^*a*^ applied.

**Working hypothesis**		**Same as (1)**	**Our choices^a^**
Parents	Total	0.35 (0.004)	−0.32 (0.013)
	Inattention	0.36 (0.009)	−0.31 (0.036)
	Hyperactivity	0.26 (0.004)	−0.24 (0.02)
Teachers	Total	0.15 (0.20)	−0.11 (0.37)
	Inattention	0.06 (0.70)	−0.17 (0.16)
	Hyperactivity	0.17 (0.13)	−0.022 (0.85)

*SEs and their corresponding p-value (in parenthesis) are presented. With the meta-analysis package, a negative SE is in favor of NFB unlike Cortese et al. (1)*.

a *Post-test values for Arnold et al. (58) are obtained after 40 sessions of NFB and Conners scale is used for Steiner et al. (57) teachers' outcomes*.

**Figure 2:**
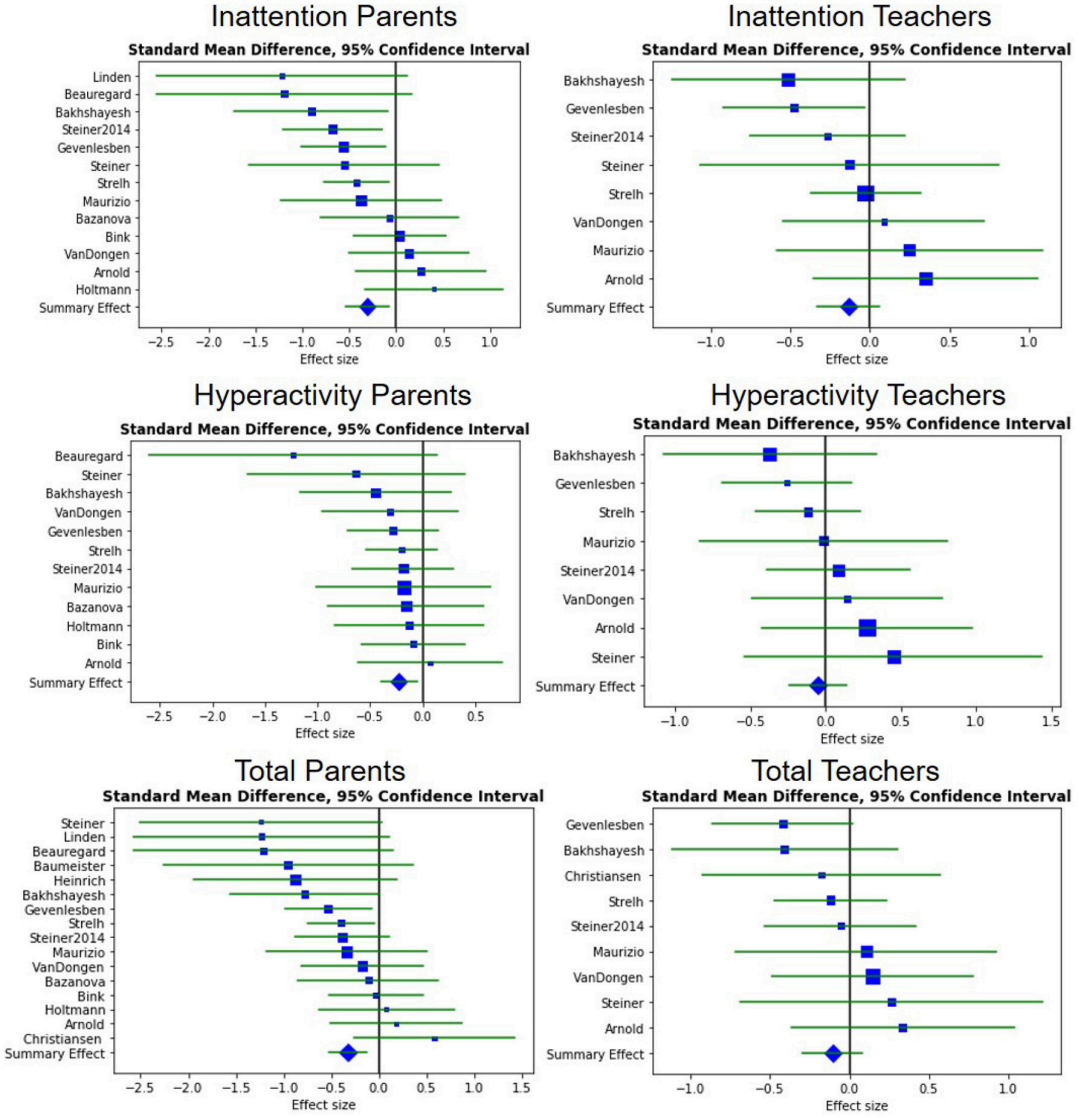
Forest plots showing the between-ES. A negative ES is in favor of NFB. The blue squares correspond to the ES, the blue diamond to the SE and the green line to the 95% confidence interval.

The meta-analysis was then extended by adding three new articles (51, 70, 71). Bazanova et al. (51) gave parents' assessments for all outcomes, Baumeister et al. (71) provided results solely for parents' total outcome, whereas Strehl et al. (70) gave both teachers' and parents' assessments for all outcomes. To be consistent with the SAOB, only the standard NFB group of Bazanova et al. (51) was included in this update. Despite favorable results for NFB, particularly on parents' assessments, adding these three new studies did not change either the magnitude or the significance of the SE, for any outcome regardless of the raters, as illustrated in Figure 2.

Regarding the “standard protocol” subgroup, Cortese et al. (1) found all the outcomes significant except for the hyperactivity symptoms rated by teachers, which showed only a statistical trend (*p*-value = 0.11). Similar results were obtained when adding the most recent studies meeting this definition (70, 71) (*p*-value = 0.11). The SE for the total outcome assessed by teachers remained significant with the addition of the new RCT (*p*-value = 0.043), thus giving more strength to this result since it is now based on four studies including 283 patients in total.

As for the no-drug subgroup, SEs were found significant for the inattention symptoms assessed by parents (*p*-value = 0.017). In addition, the differences in Arnold et al. (58) values and the inclusion of Bazanova et al. (51) caused a loss of significance in hyperactivity outcome for parents (*p*-value = 0.062) compared to Cortese et al. (1) (*p*-value = 0.016). Only Bazanova et al. (51) was included in this subgroup: in the two other studies the subjects were taking psychostimulants during the trial.

All the clinical scales used to compute the between-ES following our choices are summarized in the Supplementary Material.

### 3.3. Factors Influencing Neurofeedback

This analysis was performed on 33 trials (corresponding to 67 observations) assessing the efficacy of NFB, as presented in Table 1. The outlier rejection removed two training groups of Bazanova et al. (51) from the analysis because their within-ES were out of the bounds. Among the 28 factors selected, nine were removed because there were too many missing observations or because they were too homogeneous: beta up in frontal areas, the use of a transfer card, the type of threshold for the discrete rewards (incremental or fixed), the EEG quality equal of 3, the presence of a control group, the individualization of the frequency bands based on the iAPF, coupling NFB with EMG-Biofeedback, the severity of ADHD symptoms at baseline, and the degree of engagement with NF intervention.

All results are presented in Table 3. These results, require careful interpretation since each technique provided slightly different results. These differences may depend on the different assumptions of the model and several other factors. Nonetheless, we are inclined to trust the findings that are consistent across methods.

**Table 3:** Results of the WLS, LASSO and decision tree.

Independent variables (factors)		**Coefficients found by WLS (*p*-value)**	**Coefficients found by LASSO**	**Place on the decision tree**
Methodological	PBLIND	**0.12 (0.044)**	**0.12**	**root node**
	Randomization	0.15 (0.062)	**0.044**	/
	IRB	**–0.25 (0.01)**	0.00	/
Population	Age max	–0.13 (0.075)	0.00	/
	Age min	0.025 (0.76)	0.00	/
	On drugs	–0.091 (0.29)	0.00	/
NFB implementation	Number of sessions	**-0.36 (0.00)**	0.00	**2**^**nd**^ **node**
	Session length	**–0.34 (0.001)**	0.00	/
	Treatment length	**0.35 (0.00)**	**0.065**	**2**^**nd**^ **and 3**^**rd**^ **nodes**
	Session pace	–0.058 (0.33)	**–0.0043**	**4**^**th**^ **node**
	SMR	-0.10 (0.13)	0.00	/
	Beta up central	–0.093 (0.44)	0.00	/
	Theta down	0.043 (0.72)	0.00	/
	SCP	–0.026 (0.85)	0.00	/
	Transfer phase	**0.44 (0.00)**	0.00	/
Quality of acquisition	More than one active electrode	**–0.17 (0.010)**	**–0.033**	/
	EEG quality 2	**–0.18 (0.033)**	**–0.032**	**3**^**rd**^ **node**
Signal quality	EOG rejection or correction	**–0.35 (0.001)**	0.00	/
	Amplitude-based artifact rejection	0.052 (0.52)	0.00	/

*For the WLS, a p-value < 0.05 (in bold) means that the coefficient of the corresponding factor is significantly different from 0. For the LASSO, factors not set to 0 (in bold) are selected. For the decision tree, the place of the factor in the tree is indicated. For the first two columns, when the value of the coefficient is negative, the corresponding factor may lead to better NFB results*.

**Figure 3:**
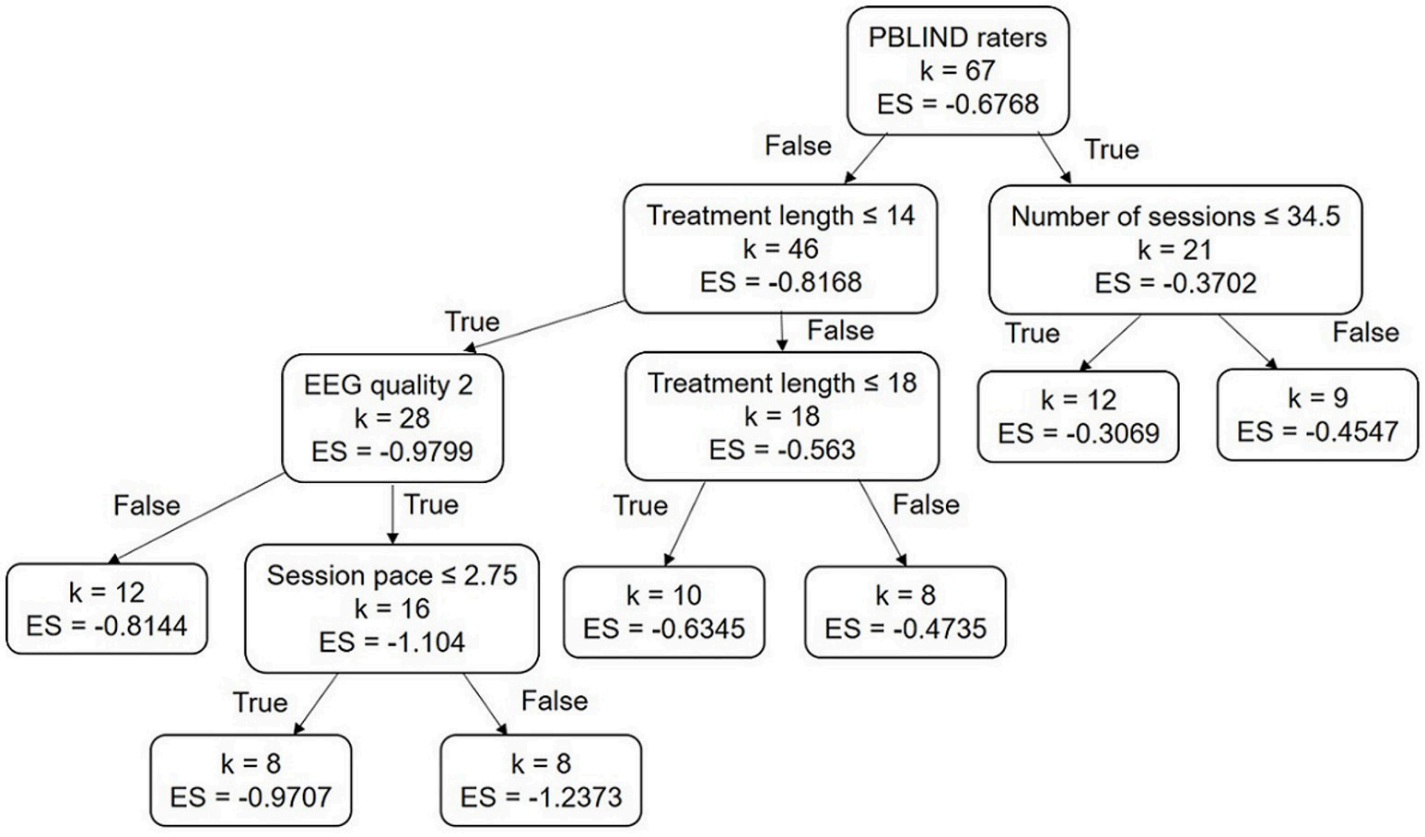
Decision Tree obtained: ES corresponds to the within subject effect size and *k* to the number of studies, the importance of variables decreases from the root node. Session length is measured in minutes, treatment length in weeks, and age in years.

The WLS technique identified nine significant factors for an adjusted R-squared of 0.62 (see second column of Table 3). When applying the OLS, the same factors were significant (except the EEG quality equal of 2 and the presence of more than one active electrode) with a lower adjusted R-squared (0.35). The LASSO regression selected six significant factors (see third column of Table 3). With these methods, a negative coefficient means that the factor is in favor of the efficacy of NFB. The decision tree is presented in Figure 3: the best predictor in our case was the PBLIND (see last column of Table 3). Four other factors also split the subsets; however, increasingly fewer samples are available the lower we get into the tree, making the interpretation increasingly doubtful.

Several factors were common to the three methods used. These included, in particular: the assessment by a blind rater, the treatment length, and an EEG quality score equal to 2 (see lines 1, 10, and 20 of Table 3). The methods also agreed on the direction of the effect for these factors: a shorter treatment and recording of the EEG with a good-quality system appears preferable, whereas teachers' assessment appears less favorable compared to parents' assessment.

It is more doubtful the influence of the factors returned by only one or two methods (see lines 2, 3, 7, 9, 12, 17, 18, and 21 of Table 3). In particular:

- both WLS and LASSO found that using more than one active electrode during NFB appears to lead to an higher efficacy;
- both WLS and the decision tree found that performing a higher number of sessions seems to be preferable;
- both LASSO and the decision tree found that a higher number of sessions per week appears to positively influence the efficacy of the NFB treatment.

Five factors were returned by only one of the methods: randomizing the groups, the IRB approval, the session length, the presence of a transfer phase, and the correction or rejection of ocular artifacts.

Eight factors were never selected by the three methods (see lines 4, 5, 6, 13, 14, 15, 16, and 23 of Table 3): the children's minimum and maximum age, being on drugs during NFB treatment, the protocols SMR, beta up in central areas, theta down, and SCP, and the artifact correction based on amplitude.

Thus, these factors overwhelmingly appear not to have an influence on NFB efficacy.

In the next section we discuss only the factors that were selected by at least two of the three methods.

## 4. Discussion

### 4.1. Meta-Analysis

This replication and update of a meta-analysis did not meet all PRISMA recommendations (107). In particular, the risk of bias in individual and across studies was not assessed.

In the meta-analysis performed here, we challenged some choices made by Cortese et al. (1) which proved controversial: the computation of between-ES based on an unusual scale (57) and the inclusion of a pilot study (58) whose endpoint values were not available at the time Cortese et al. (1) conducted their meta-analysis. We here review the list of changes, their justification, and their impact on the analysis.

First, relying on the Conners-3 (108) instead of the BOSS Classroom Observation (72) for teachers' ratings seems preferable because this scale is more commonly used (74, 75) and is a revision of the Conners Rating Scale Revised (73) whose reliability has been studied (76). However, relying on one or the other scale did not change the significance of the between-ES, regardless of outcome.

Second, to compute the between-ES of Arnold et al. (58) the clinical scores taken when all sessions were completed were used instead of looking at interim results as with Cortese et al. (1). Some studies suggested that the number of sessions correlates positively with the changes observed in the EEG (61) so that a lower number of sessions would lead to artificially smaller between-ES. Here, the between-ES computed with the values at post test of Arnold et al. (58) were smaller than those obtained after 12 sessions; however, these differences did not lead to a change of significance of the SE.

To conclude on this meta-analysis, although some points were controversial, the impact on the meta-analysis was minimal and did not change the statistical significance of any outcome. The addition of the three new studies (51, 70, 71) further confirmed the original results. Indeed, the significance did not change for any outcome: the SE remained significant for MPROX raters and non-significant for PBLIND. Adding three more studies increased the significance of the sensitivity analysis run by Cortese et al. (1) most notably the SE of studies corresponding to NFB “standard protocols” (59). While Cortese et al. (1) found that this subset tends to perform better, particularly on the PBLIND outcome, adding two studies confirmed this result on the total clinical score (*p*-value < 0.05). Despite the obvious heterogeneity of the studies included in this subset (particularly in terms of protocol used), these results suggest a positive relation between the features of this *standard* design and NFB performance. This result is a breakthrough in the demonstration of standard NFB protocol efficacy for the treatment of ADHD. Nonetheless, the studies included in this subset are still highly heterogeneous (particularly in terms of protocol used), a factor which should be accounted for.

### 4.2. Factors Influencing Neurofeedback

Description and analysis of different types of NFB implementation were subject to several studies (1, 54, 59–62). However, to the best of our knowledge, none of these studies has implemented a systematic multivariate approach to associate factors to clinical endpoints therefore exposing their univariate analysis to a greater extend to the presence of a confounding factor.

Two observations were detected as outliers and so removed from the dataset before performing the SAOB: Bazanova et al.'s (51) individualized NFB and individualized NFB coupled with EMG-Biofeedback groups. Indeed, these two groups presented very large within-ES (–3.41 and –3.95), even bigger than those reported in the literature on medication treating ADHD (109). These large values broke our working hypothesis, so in order to be able to conclude on the results obtained by the SAOB, an outlier rejection was implemented.

As expected, the number of sessions was found to be significant, even if it was by only two methods, which was in compliance with existing literature. For instance, using several univariate regressions without correction for multiple testing (54), Arns et al. (59) stated that performing less than 20 NFB sessions leads to smaller effects. Similarly, Vernon et al. (61) observed that positive changes in the EEG and behavioral performance occurred after a minimum of 20 sessions. However, Enriquez-Geppert et al. (60) insisted that the number of sessions should be carefully chosen in order to avoid “overtraining.” The fact that the number of sessions was not identified by the LASSO as a positively contributing factor might be explained by the presence of only two data points with 20 sessions or less. Conceivably, the temporal threshold of efficacy was passed for all included studies, making the identification of this factor by the three methods unlikely on this dataset. However, the two methods that identified this factor both agreed that as expected, the more sessions performed, the more efficient the NFB tends to be.

Interestingly, Minder et al. (110) suggests that the subject location of the NFB training may also be an important contributory factor to clinical effectiveness. However, this has been challenged by a recent study (110) showing that performing NFB at school or at the clinic has no significant impact on treatment response.

The type of NFB protocol was not identified by any method, and did not appear to influence the NFB results. This minimal importance granted by the methods to the NFB protocols is counter-intuitive given the centrality of the protocols in the neurophysiological mode of action and subsequent expected impact on therapeutic effectiveness (61). A possible explanation for this result is that these protocols were equally efficacious for the populations to whom they were offered and thereby did not constitute a significant explanatory factor. This result, however, does not preclude a combined and personalized strategy (offering personalized protocols based on phenotypes) to further improve performance, as previously suggested by Alkoby et al. (111).

Several factors were selected by all three methods with the same direction of influence: the EEG quality, the treatment length, and the rater's probably blindness to the treatment. First, our analysis highlighted that recording EEG in good conditions leads to better results. This can be explained by the fact that better signal quality enables more accurate extraction of EEG patterns linked to ADHD and hence leads to better learning and therapeutic efficacy (63). However, it remains difficult to assess the quality of EEG hardware (such as the amplifier used) because little information is provided in these studies. This calls for greater care in future studies, which should strive to assess and report the quality of the data.

Next, it appears that the longer the course of treatment, the less efficient it becomes. This may be explained by the degree of engagement with NFB intervention: it may be harder to be engaged with a long course of treatment. However, it is difficult to quantify because either no questionnaires assessing engagement were submitted to children or this information was not provided. It is an interesting point to investigate, so we invite future studies to share it if possible.

Arguably, the treatment length is a proxy for treatment intensity, suggesting that a shorter period of treatment is more likely to succeed because the frequency of the sessions is higher. This hypothesis is supported by the fact that the variable *session pace* (number of sessions per week) is also associated with larger within-ES according to the LASSO and the decision tree. The impact of the intensity of treatment has been investigated by Rogala et al. (112) on healthy adults: it was observed that studies with at least four training sessions completed on consecutive days were all beneficial. Overall, these results suggest adopting a high session pace, which is not common knowledge in the field.

Some other factors' influence would have been interesting to investigate, such as using personalized NFB protocols based on the iAPF (49), which seems promising according to Bazanova et al. (51) and Escolano et al. (50). However, it could not be included in the SAOB because only two studies used personalized NFB protocols. This lack of studies is also the reason why the impact of coupling EMG-Biofeedback with NFB could not be included in the SAOB. Another interesting factor, which could have helped explain the result on the treatment duration, was excluded from the analysis: the severity at baseline. Although pre-test scores were available for each study, they could not be compared because they were measured on different scales. To address this problem, the scores were normalized using the maximum score to be obtained on each scale. However, this value was not found for several clinical scales, which led to missing observations. When more studies including these features will be available, it would be interesting to run the SAOB to determine the influence of these excluded factors.

In general our results strongly support the effectiveness of NFB for the treatment of ADHD. However, as expected, the assessment of symptoms by non-blind raters leads to far more favorable results than by PBLIND raters, a result widely expected and in close compliance with the existing meta-analysis (1, 55). This observation would certainly be contradictory should teachers' assessments reflect a placebo effect, which has long been documented in the literature (110, 113, 114). This point is investigated in greater detail in the following section.

### 4.3. Analysis on the Probably Blind Raters

**Figure 4:**
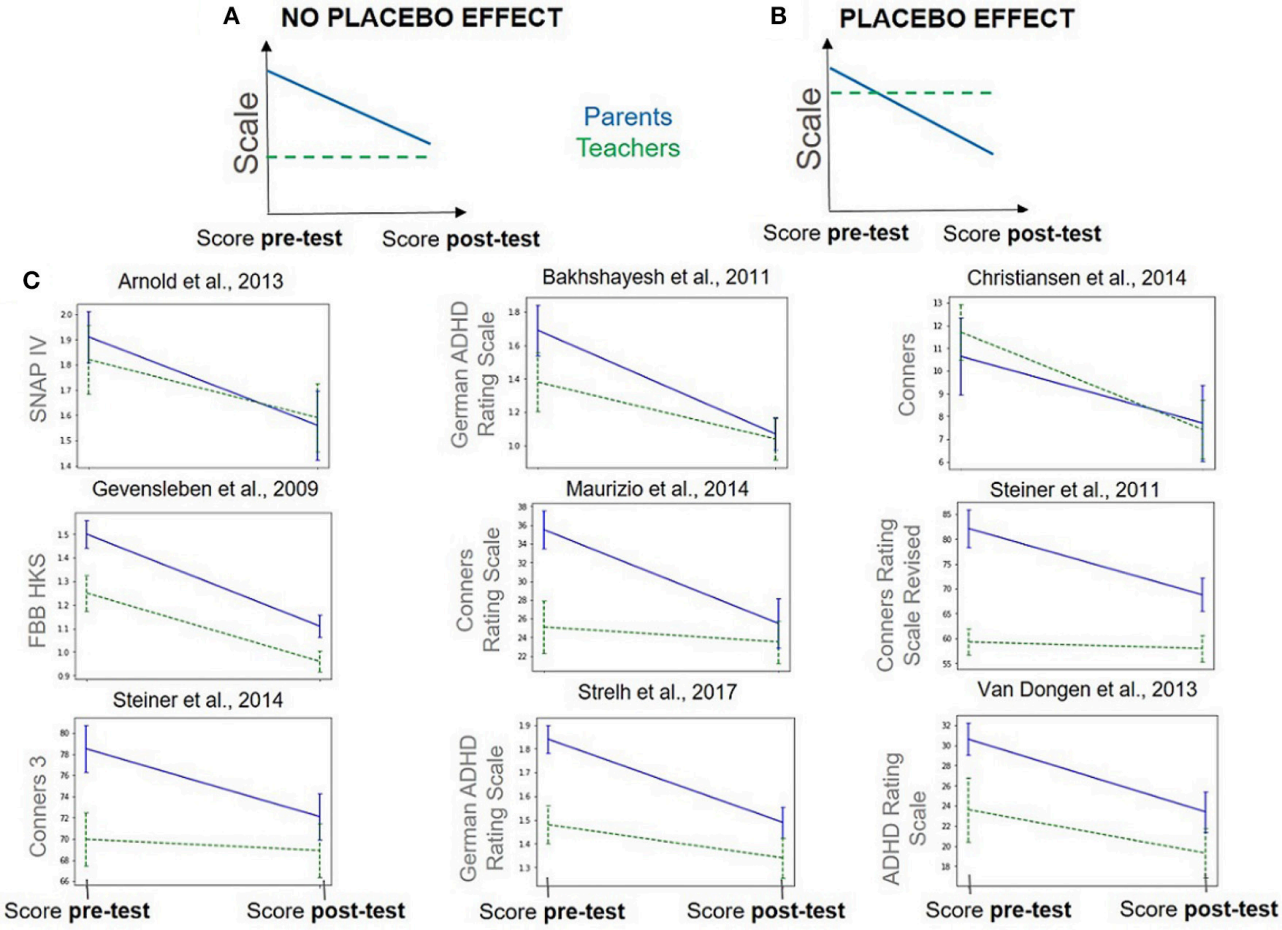
Pre-test and post-test scores (± standard error) given by parents (MPROX) in blue and teachers (PBLIND) in green, dashed line. Data hypothesized under two different hypotheses: **(A)** no placebo effect, teachers see fewer symptoms altogether so that difference pre-post is low and **(B)** placebo effect, teachers see as many baseline symptoms as parents but do not see as much improvement. **(C)** Real data: evolution of parents' and teachers' scores between pre- and post-test on studies that satisfy Cortese et al.'s (1) inclusion criteria and that provide teachers and parents' scores on the same scale.

Teachers were considered as PBLIND raters by Cortese et al. (1) and Micoulaud-Franchi et al. (55). Unexpectedly, the data provided did not exactly match the widely accepted hypothesis stating that the difference between MPROX and PBLIND can solely be explained by the placebo effect. Nonetheless, the emphasis put on 'probably' indicates that teachers may be aware of the treatment followed. An element that corroborates this hypothesis is the fact that, for all the studies included in this work, the amplitude of the clinical scale at baseline suggests that teachers did not capture the full extent of the symptoms or, put differently, that they were blind more to the symptoms than to the intervention, as illustrated in Figure 4. Indeed, before the intervention, teachers rated the symptoms less severely compared to parents and observed less improvement at post-test: this tends to correspond more to case A with no placebo effect than case B. The expected differences of ratings between teachers and parents have been extensively studied (110, 113, 114), observing that teachers are more likely to underrate a child's symptom severity, especially for younger children. As a consequence, teachers might simply be less likely to observe a clinical change over the course of the treatment (110, 113, 114). Moreover, it is also clear that there is more variability in teachers' scores compared to parents', which could partly explain the lower ES obtained for PBLIND raters, since the variability deflates the ES. In conclusion, using PBLIND as an estimate for correcting the placebo effect does not appear an appropriate choice.

Another way to highlight a possible placebo effect is to focus on the decision tree illustrated in Figure 3. The top node splits: on the one hand 46 observations corresponding to MPROX raters and, on the other, 21 observations corresponding to PBLIND. If the differences observed between PBLIND and MPROX raters were due to the placebo effect, one would expect to find in the MPROX sub-tree some factors linked to the perception of the implication in the treatment. This was indeed the case: treatment length was found to be significant but not in the direction corroborating the hypothesis that they are a part of a placebo effect. Indeed, one would expect that the longer the treatment, the higher the placebo effect and the greater the within-ES. Instead, the opposite was found, somewhat invalidating the hypothesis.

Overall, these results suggest that PBLIND assessments could hardly be used to assess placebo effect as they seem to be blinder to symptoms than to intervention. In the absence of an ethically (115) and technically (116) feasible sham for NFB protocols (117), it is necessary to fall back on an acceptable methodological alternative for the demonstration of clinical effectiveness. Among those are the analysis of neuromarkers collected during NFB treatment demonstrating that patients do *control* the trained neuromarkers; that they *learn* (reinforce control over time), and that these possibly lead to lasting brain reorganization (e.g., changes in their baseline resting state activity). The specificity of these changes, in relation to which neuromarkers were trained and to the clinical improvement, will be an essential component of this demonstration.

## 5. Conclusion

In this work we provide additional elements in favor of the effectiveness of NFB for the treatment of ADHD. First, we confirm that a subgroup of standard NFB studies shows a statistically significant improvement on PBLIND assessments (k = 4 studies instead of 3, *n* = 283 patients instead of 158 (1)).

Second, we identify technical factors as positive contributors to clinical effectiveness, which strongly suggests that it is mediated by a real mechanism of action based on EEG conditioning. Equally, treatment intensity was also found to contribute, corroborating what is known from learning theory (memory consolidation) (118); that is to say, a more intense treatment leads to an increased clinical efficacy.

While these findings certainly contribute to the debate, this work also suggests that the ultimate demonstration of evidence remains out of reach, as teachers' assessments were partly invalidated as a proxy for the quantification of the placebo effect. As a consequence, using PBLIND endpoints to address the specificity of the clinical efficacy is not recommended and we instead advise a reliance on other available methodological tools. These tools include sham NFB and neuromarker analysis investigating the specificity of the EEG changes with respect to trained neuromarkers as well as changes in clinical endpoints.

This work also offers an open-source toolbox for running meta-analysis and SAOB: the code and data used are available, thus ensuring the transparency and replicability of these analysis, as well as fostering future ones. Regarding perspectives, this two-fold methodological framework applied to NFB for ADHD could be suitable for other NFB applications (29).

## Data Availability

The datasets analyzed for this study can be found in the repository (64) https://github.com/AuroreBussalb/meta-analysis-statistical-tools.

## Author Contributions

AB extracted all data from articles and performed the analysis. MC provided advice and expertise concerning the design of the study and the methods used. RD and EA provided clinical interpretation of the results. QB and DO supported the toolbox implementation and validation. LM supervised the entirety of this work.

### Conflict of Interest Statement

AB, QB, DO, and LM work for Mensia Technologies. MC served as scientific advisor for Mensia Technologies when this work was conducted. The remaining authors declare that the research was conducted in the absence of any commercial or financial relationships that could be construed as a potential conflict of interest.

## Footnotes

^1^Baseline severity is given by the pre-treatment score. But, to have comparable severity scores between the different clinical scales, pre-treatment score is normalized, i.e., divided by the maximal score which can be obtained on the clinical scale.

## Abbreviations

ADHD: Attention Deficit/Hyperactivity Disorder
AgCl: Silver Chloride
Au: Gold
CPT: Continuous Performance Test
EEG: electroencephalogram
EMG: Electromyogram
EOG: Electro-Oculogram
ERP: Event-Related Potential
ES: Effect Size
FDA: Food and Drug Administration
fMRI: functional Magnetic Resonance Imaging
iAPF: individual Alpha Peak Frequency
IRB: Institutional Review Board
LASSO: Least Absolute Shrinkage and Selection Operator
MPROX: Most Proximal
MSE: Mean Square Error
NFB: neurofeedback
OLS: Ordinary Least Squares
PBLIND: Probably Blind
PET: Positron Emission Tomography
RCT: Randomized Controlled Trial
SAOB: Systematic Analysis of Biases
SART: Sustained Attention to Response Task
SCP: Slow Cortical Potential
SE: Summary Effect
SMR: Sensorimotor Rhythm
TBR: Theta Beta Ratio
TOVA: Test of Variables of Attention
WLS: Weighted Least Squares.
