# Supplementary material for: Clinical and Experimental Factors Influencing the Efficacy of Neurofeedback in ADHD: A Meta-Analysis
Source: Front Psychiatry. 2019 Feb 18;10:35. doi: 10.3389/fpsyt.2019.00035 (PMC6388544; doi:10.3389/fpsyt.2019.00035)
Supplement: Supplementary file 1 [file Data_Sheet_1.PDF]

Supplementary Material: Clinical and experimental factors influencing the efficacy of neurofeedback in ADHD: a meta-analysis

1 SUPPLEMENTARY MATERIALS AND METHODS

1.1 Studies selection

Search terms entered in Pubmed (last check on 12/02/2018) were: «(ADHD OR adhd OR attention deficit disorder with hyperactivity OR minimal brain disorders OR syndrome hyperkinetic OR hyperkinetic syndrome OR hyperactivity disorder OR hyperactive child syndrome OR childhood hyperkinetic syndrome OR attention deficit hyperactivity disorders OR attention deficit hyperactivity disorder OR adhd attention deficit hyperactivity disorder OR addh OR overactive child syndrome OR attention deficit hyperkinetic disorder OR hyperkinetic disorder OR attention deficit disorder hyperactivity OR attention deficit disorders hyperactivity OR child attention deficit disorder OR hyperkinetic syndromes OR syndromes hyperkinetic OR hyperkinetic syndrome childhood) AND (randomized control trial OR RCT OR randomized control study OR Pilot Study OR Study OR Trial OR randomized trial) AND (neurofeedback OR "EEG biofeedback" OR neurotherapy OR SCP OR "slow cortical potentials OR Theta Beta Ratio or TBR)"».

1.2 Perform a meta analysis

To conduct meta-analysis, different software exist: for instance Cortese et al. (2016) used RevMan 5.3 (Cochrane Collaboration, 2011) that computes the between-Effect Size (ES) and its variance of each included study by applying the formula presented in Morris (2008). However, in order to compute the variance of the between-ES, the pooled within-group Pearson correlation r (*i.e.* the pre-post correlation) is required (James et al., 2013). In our case, this correlation was not known and the raw data were not available so we took an approximation: Balk et al. (2012) found that a value of 0.5 yields values close to those computed with the actual value of the correlation.

In this replication of the work of Cortese et al., the same formulas were used (Borenstein et al., 2009), but instead of using RevMan, a Python code was developed in order to perform the meta-analysis. To increase replicability and transparency and promote open science, we provide the full raw data used for this research as well as the Python code on a GitHub repository (Bussalb, 2019); it was tested with Cortese et al. (2016) raw data to verify that the same results were found.

To perform the meta-analysis several steps must be followed. First the choice of the model: this analysis is based on either one of the following statistical models (Borenstein et al., 2009):

- *the fixed-effect model*: the true between-ES (i.e. the between-ES that would be observed with an infinitely large sample size) is the same for all the studies in the analysis. The differences between the actually observed between-ES are due to sampling errors;
- *the random-effects model*: the true between-ES could vary from study to study. The differences between the observed ESs are due to sampling errors but also to the various designs of the studies (for instance the number of participants or the implementation).

In the present case, although the studies included into the meta-analysis met the same criteria, they remained different from each other on various aspects, so the random effects model is more appropriate than the fixed-effect model.

1.3 Compute the between effect size of each study

First, the scores presented in the articles were extracted and the between-ES of each study as defined in Morris (2008) was computed:

$$\text{ES} = c_p \left(\frac{(M_{\text{post},T} - M_{\text{pre},T}) - (M_{\text{post},C} - M_{\text{pre},C})}{\sigma_{\text{pre}}} \right). \tag{S1}$$

An between-ES is equivalent to a z-score of a standard normal distribution. It is computed as mean pre- to post-treatment score change in the neurofeedback (NFB) group ($M_{\text{pre},T}$, $M_{\text{post},T}$) minus the mean pre- to post- treatment score change in the control group ($M_{\text{pre},C}$, $M_{\text{post},C}$), divided by the pooled pre-test standard deviation (σ_{pre}):

$$\sigma_{\text{pre}} = \sqrt{\frac{(n_T - 1)\sigma^2_{\text{pre},T} + (n_C - 1)\sigma^2_{\text{pre},C}}{n_T + n_C - 2}}, \tag{S2}$$

where $\sigma_{t,G}$ indicates the standard deviation for group G at time t and n_G defines the sample size of each group; c_p is a bias adjustment typically used for small sample sizes:

$$c_p = 1 - \frac{3}{4(n_T + n_C - 2) - 1}. \tag{S3}$$

1.4 Compute the variance of each between effect size

Then, the variance of each between-ES was computed (Morris, 2008):

$$\sigma^2(\text{ES}) = c_p^2 \left(\frac{n_T + n_C - 2}{n_T + n_C - 4} \right) \left(\frac{2(1-r)(n_T + n_C)}{n_T n_C} + \text{ES}^2 \right) - \text{ES}^2. \tag{S4}$$

To compute the variance of the between-ES, the pooled within-group Pearson correlation r (*i.e.* the pre-post correlation) is required (James et al., 2013):

$$r = \frac{\sum_{i=1}^{n} (\text{pre}_i - \mu_{\text{pre}})(\text{post}_i - \mu_{\text{post}})}{\sqrt{\sum_{i=1}^{n} (\text{pre}_i - \mu_{\text{pre}})^2} \sqrt{\sum_{i=1}^{n} (\text{post}_i - \mu_{\text{post}})^2}}, \tag{S5}$$

where n is the number of patients included in a study, pre_i, post_i are score values for patient i at pre- and post-test respectively, and μ_{pre}, μ_{post} the mean scores over all patients. It is a measure of linear correlation between two variables. A value of 1 means that there is a positive correlation whereas a value of -1 means a negative correlation. When $r = 0$, there is no linear correlation. In our case, this correlation was not known and the raw data were not available so we took an approximation following Balk et al. (2012), who found that a value of 0.5 yielded values close to those computed with the actual value of the correlation.

Once variances were obtained with Eq. (S4), we could compute the standard error and the 95% confidence interval of each between-ES.

1.5 Compute the weight of each study

To compute the Summary Effect (SE) a weight must be assigned to each study. To obtain them several steps must be followed. At first, the fixed-effects model weight w_{fixed_k} of each study k was computed as defined in Borenstein et al. (2009):

$$w_{\text{fixed}_k} = \frac{1}{\sigma^2(\text{ES}_k)}. \tag{S6}$$

Nevertheless, we choose to use the random effects model, so the weights ($w_{\text{random}_k} = w_k$) associated to this model are different. To compute them, the between-studies variance τ^2 is required. It was calculated in three steps described in Eq. (S7), Eq. (S8) and Eq. (S9) (Borenstein et al., 2009):

$$Q = \sum_{k=1}^{K} (w_{\text{fixed}_k} \text{ES}_k^2), \tag{S7}$$

$$C = \sum_{k=1}^{K} w_{\text{fixed}_k} - \frac{\sum_{k=1}^{K} (w_{\text{fixed}_k})^2}{\sum_{k=1}^{K} w_{\text{fixed}_k}}, \tag{S8}$$

with K the total number of included studies, and

$$\tau^2 = \frac{Q - \text{df}}{C}, \tag{S9}$$

with $\text{df} = K - 1$ the degrees of freedom.

The random-effects model takes into account the differences between the studies, so the weights are equal to the inverse of the sum between the within-study variance (the variance of the between-ES) and the between-studies variance:

$$w_k = \frac{1}{\sigma^2(\text{ES}_k) + \tau^2}. \tag{S10}$$

1.6 Compute the summary effect

Finally, the weighted average of the K between-ES was computed to obtain the SE as described in Eq. (S11) (Borenstein et al., 2009):

$$\text{SE} = \frac{\sum_{k=1}^{K} w_k \text{ES}_k}{\sum_{k=1}^{K} w_k}. \tag{S11}$$

Once the SE is obtained, we can compute its variance, its standard error, its 95% confidence interval, its p-value, and I^2 estimating between-ES's between studies heterogeneity.

1.7 Scales used for the meta-analysis

All scales used for the meta-analysis are summarized in Table S1 in order to facilitate the replication of this work.

1.8 Associate independent factors to effect sizes

Three different methods were used to perform the Systematic Analysis of Biases (SAOB):

- weighted multiple linear regression with Weighted Least Squares (WLS) (Montgomery et al., 2012);

- sparsity-regularized linear regression with Least Absolute Shrinkage and Selection Operator (LASSO) (Tibshirani, 1996);
- decision tree regression (Quinlan, 1986).

We first applied the WLS as described in Eq. (S12):

$$\mathbf{W}y = \mathbf{WX}\beta + \epsilon. \tag{S12}$$

X is a $(n \times p)$ full rank matrix and represents n observations on each $p - 1$ independent variables and an intercept term, β is a $(p \times 1)$ vector of associated regression coefficients, **W** is a $(n \times n)$ diagonal matrix with weights, y is a $(n \times 1)$ vector of dependent variables and ϵ is a $(n \times 1)$ vector of errors. The aim of the WLS is to estimate the vector of coefficients β by minimizing the Weighted Residual Sum of Squares (WRSS):

$$\text{WRSS} = \sum_{i=1}^{n} w_i \Big(y_i - \beta_0 - \sum_{j=1}^{p} \beta_j x_{ij} \Big)^2. \tag{S13}$$

A significant coefficient (meaning significantly different from 0) indicates that the associated factor has an influence on NFB efficacy and the sign of the coefficient indicates the direction of the effect.

The second method applied was the LASSO, which naturally incorporates variable selection in the linear model thanks to ℓ_1-norm applied on the coefficients. The coefficients $\hat{\beta}_j$ are obtained by minimizing the cost:

$$\hat{\beta} = \underset{\beta}{\operatorname{argmin}} \sum_{i=1}^{n} \Big(y_i - \beta_0 - \sum_{j=1}^{p} \beta_j x_{ij} \Big)^2 + \lambda \sum_{j=1}^{p} |\beta_j| \,, \tag{S14}$$

where λ is the regularization parameter setting more coefficients to zero as it increases. The optimal tuning parameter was determined by a leave-one-out cross-validation. This method retains one observation as the validation data for testing the model and the remaining n - 1 observations are used as training data. The cross-validation process is then repeated n times with each of the observation used exactly once as the testing data. For each fold, the Mean Square Error (MSE) on the test set was computed and eventually the n results can be averaged to produce a single observation that enables to find the optimal λ: it corresponds to the abscissa of the minimum value of the MSE on the mean fold computed for a large range of λ (James et al., 2013). A coefficient not set at zero means that the associated factor may have an influence on NFB and once again, the sign of the coefficient indicates the direction of the effect.

The last method used to determine factors influencing NFB was the decision tree (Quinlan, 1986), a non linear method. It brakes down a dataset into smaller and smaller subsets using at each iteration a variable and a threshold chosen to optimize a simple MSE criterion:

$$\text{MSE} = \frac{1}{n} \sum_{i=1}^{n} \Big(\hat{y}_i - y_i \Big)^2, \tag{S15}$$

with $\hat{y}$ the predicted values.

2 SUPPLEMENTARY RESULTS

2.1 Perform a meta-analysis

First, when using the between-ES found by Cortese et al. (2016) thanks to RevMan (Cochrane Collaboration, 2011), and then performed the following steps of meta-analysis with the Python code, we observed no major differences between these results and those obtained with RevMan (Cochrane Collaboration, 2011) as listed in Table S2. The sign difference is explained by our choice that a negative between-ES is in favor of NFB. The minor discrepancies, especially observed at the p-values level, were due to our choice to always use a pre-post correlation of 0.5 when computing the variance of each between-ES. Moreover, a sensitivity analysis was conducted to ensure the minor impact of the pre-post correlation value: when it varied between 0.2 and 0.8 the significance of the SE did not change.

Thanks to the previous step, we could conclude that the Python code yielded results close to those returned by RevMan Cochrane Collaboration (2011).

2.2 Detect factors influencing the Neurofeedback

To assess the variability of each factor, box plots of their standardized values were displayed in Figure S1: the variability in treatment and sessions length as well as the number of sessions is more important between studies than the number of sessions per week and the age bounds.

2.3 Detect factors influencing the Neurofeedback

To assess the variability of each factor, box plots of their standardized values were displayed in Figure S1: the variability in treatment and sessions length as well as the number of sessions is more important between studies than the number of sessions per week and the age bounds.

2.4 Assumptions for applying linear regression

The first method used to detect the influencing factors was the WLS. The assumptions inherent to this method were checked:

- the moment matrix $\mathbf{X}^T\mathbf{W}^T\mathbf{W}\mathbf{X}$ is non-singular;
- no apparent correlation between the continuous independent variables is found;
- the fit is significant as shown by the F-statistic (p = 7.58e-08);
- the residuals are normally distributed as demonstrated by the skew (0.337), kurtosis (3.154) and the Omnibus test (prob(Omnibus) = 0.392).

These assumptions were also satisfied for the Ordinary Least Squares (OLS).

2.5 Figures

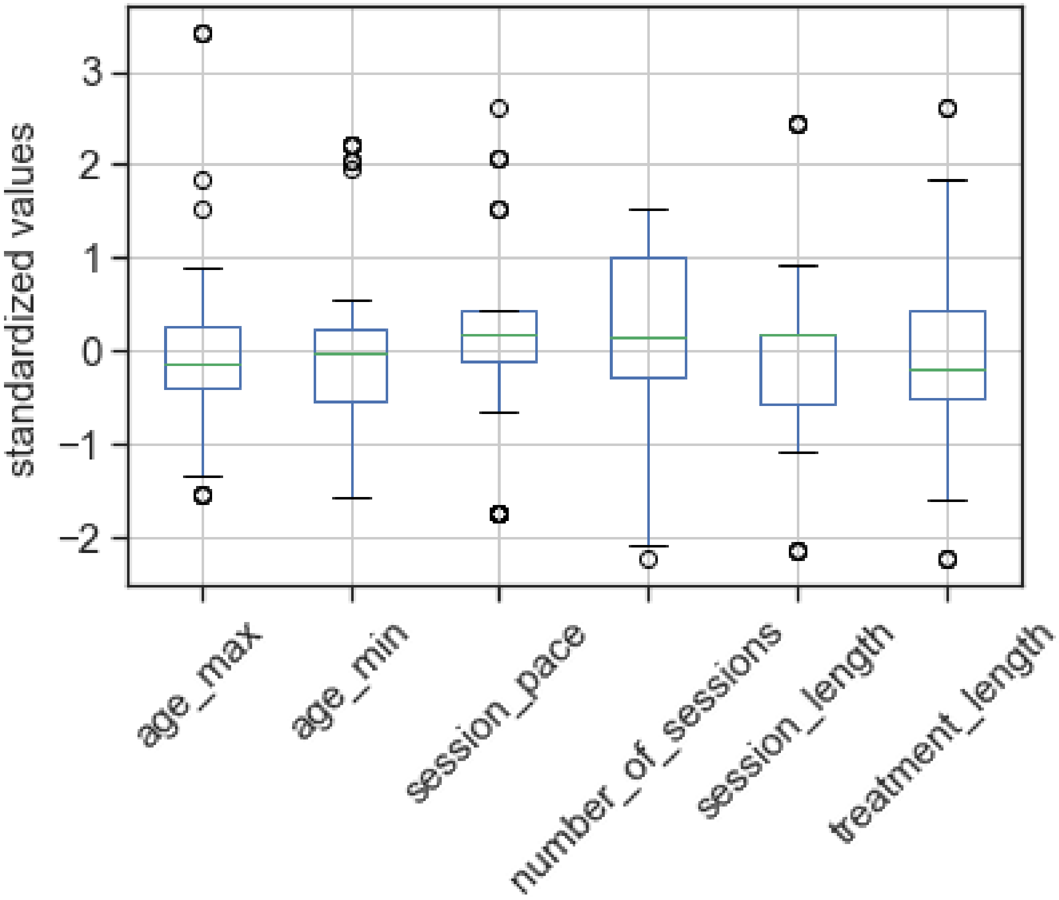

Figure S1. Boxplots of the standardized values of each continuous factor.

2.6 Tables

Table S1. Clinical scales used to update Cortese et al. (2016) with our choices and the two new articles.

Study	Outcome	Score Names - Parents ratings	Score Names - Teachers ratings
Arnold et al.	Total	SNAP IV	SNAP IV
	Inattention	SNAP IV	SNAP IV
	Hyperactivity	SNAP IV	SNAP IV
Bakhshayesh et al.	Total	German ADHD-RS	German ADHD-RS
	Inattention	German ADHD-RS	German ADHD-RS
	Hyperactivity	German ADHD-RS	German ADHD-RS
Baumeister et al.	Total	DISYPS	-
Bazanova et al.	Total	SNAP IV	-
	Inattention	SNAP IV	-
	Hyperactivity	SNAP IV	-
Beauregard and Levesque	Total	CPRS	-
	Inattention	CPRS	-
	Hyperactivity	CPRS	-
Bink et al.	Total	ADHD-RS self report	-
	Inattention	ADHD-RS self report	-
	Hyperactivity	ADHD-RS self report	-
Christiansen et al.	Total	Conners-3 Parents	Conners-3 Teachers
Gevensleben et al.	Total	German ADHD-RS	German ADHD-RS
	Inattention	German ADHD-RS	German ADHD-RS
	Hyperactivity	German ADHD-RS	German ADHD-RS
Heinrich et al.	Total	German ADHD-RS	-
Holtmann et al.	Total	German ADHD-RS	-
	Inattention	German ADHD-RS	-
	Hyperactivity	German ADHD-RS	-
Linden et al.	Total	IOWA Conners	-
	Inattention	IOWA Conners	-
Maurizio et al.	Total	CPRS	CTRS
	Inattention	CPRS	CTRS
	Hyperactivity	CPRS	CTRS
Steiner et al.	Total	Conners Rating Scales Revised	Conners Rating Scales Revised
	Inattention	Conners Rating Scales Revised	Conners Rating Scales Revised
	Hyperactivity	Conners Rating Scales Revised	Conners Rating Scales Revised
Steiner et al.	Total	Conners-3 Parents	Conners-3 Teachers
	Inattention	Conners-3 Parents	Conners-3 Teachers
	Hyperactivity	Conners-3 Parents	Conners-3 Teachers
Strehl et al.	Total	German ADHD-RS	German ADHD-RS
	Inattention	German ADHD-RS	German ADHD-RS
	Hyperactivity	German ADHD-RS	German ADHD-RS
van Dongen-Boomsma et al.	Total	ADHD RS	ADHD RS
	Inattention	ADHD RS	ADHD RS
	Hyperactivity	ADHD RS	ADHD RS

SNAP: Wanson, Nolan and Pelham Questionnaire, ADHD-RS: ADHD Rating Scale, CPRS: Conners Parent Rating Scale, CTRS: Conners Teacher Rating Scale, BOSS Classroom Observation: Behavioral Observation of Students in Schools, DISYPS: Diagnostic System of Mental Disorders in Children and Adolescents.

Table S2. Comparison between Cortese et al. (2016) results obtained with RevMan (Cochrane Collaboration, 2011) and those obtained with the Python code. Summary effects and their corresponding p-value in parenthesis are presented. With the Python program, a negative summary effect is in favor of NFB.

Input data		Results from Cortese et al. (2016)	Effect sizes from Cortese et al. (2016)
Implementation		RevMan	meta-analysis package
	Total	0.35 (0.004)	-0.34 (0.004)
Parents	Inattention	0.36 (0.009)	-0.35 (0.011)
	Hyperactivity	0.26 (0.004)	-0.24 (0.02)
	Total	0.15 (0.20)	-0.13 (0.25)
Teachers	Inattention	0.06 (0.70)	-0.09 (0.50)
	Hyperactivity	0.17 (0.13)	-0.15 (0.21)

REFERENCES

Arnold, L. E., Lofthouse, N., Hersch, S., Pan, X., Hurt, E., Bates, B., et al. (2014). Eeg neurofeedback for attention-deficit/hyperactivity disorder: Double-blind sham-controlled randomized pilot feasibility trial. *Journal of Atention Disorder* doi:10.1177/1087054712446173

Bakhshayesh, A. R., Hänsch, S., Wyschkon, A., Rezai, M. J., and Esser, G. (2011). Neurofeedback in adhd: a single-blind randomized controlled trial. *European child & adolescent psychiatry* 20, 481. doi:10.1007/s00787-011-0208-y

Balk, E. M., Earley, A., Patel, K., Trikalinos, T. A., and Dahabreh, I. J. (2012). Empirical assessment of within-arm correlation imputation in trials of continuous outcomes

Baumeister, S., Wolf, I., Holz, N., Boecker-Schlier, R., Adamo, N., Holtmann, M., et al. (2016). Neurofeedback training effects on inhibitory brain activation in adhd: A matter of learning? *Neuroscience* doi:10.1016/j.neuroscience.2016.09.025

Bazanova, O. M., Auer, T., and Sapina, E. A. (2018). On the efficiency of individualized theta/beta ratio neurofeedback combined with forehead emg training in adhd children. *Frontiers in human neuroscience* 12, 3

Beauregard, M. and Levesque, J. (2006). Functional magnetic resonance imaging investigation of the effects of neurofeedback training on the neural bases of selective attention and response inhibition in children with attention-deficit/hyperactivity disorder. *Applied psychophysiology and biofeedback* 31, 3–20. doi:10.1007/s10484-006-9001-y

Bink, M., van Nieuwenhuizen, C., Popma, A., Bongers, I. L., and van Boxtel, G. J. M. (2014). Behavioral effects of neurofeedback in adolescents with adhd: a randomized controlled trial. *European Child and Adolescent Psychiatry* doi:10.1007/s00787-014-0655-3

Borenstein, M., Hedges, L. V., Higgins, J., and Rothstein, H. R. (2009). *Introduction to meta-analysis* (Wiley)

[Dataset] Bussalb, A. (2019). Meta-analysis statistical tools. `https://github.com/AuroreBussalb/meta-analysis-statistical-tools`

Christiansen, H., Reh, V., Schmidt, M. H., and Rief, W. (2014). Slow cortical potential neurofeedback and self-management training in outpatient care for children with adhd: study protocol and first preliminary results of a randomized controlled trial. *Frontiers in human neuroscience* 8. doi:10.3389/fnhum.2014.00943

[Dataset] Cochrane Collaboration (2011). Revman 5.1. `https://community.cochrane.org/help/tools-and-software/revman-5`

Cortese, S., Ferrin, M., Brandeis, D., Holtmann, M., Aggensteiner, P., Daley, D., et al. (2016). Neurofeedback for attention-deficit/hyperactivity disorder: meta-analysis of clinical and neuropsychological outcomes from randomized controlled trials. *Journal of the American Academy of Child & Adolescent Psychiatry* 55, 444–455. doi:10.1016/j.jaac.2016.03.007

Gevensleben, H., Holl, B., Albrecht, B., Vogel, C., Schlamp, D., Kratz, O., et al. (2009). Is neurofeedback an efficacious treatment for adhd? a randomised controlled clinical trial. *Journal of Child Psychology and Psychiatry* 50, 780–789. doi:10.1111/j.1469-7610.2008.02033.x

Heinrich, H., Gevensleben, H., Freisleder, F. J., Moll, G. H., and Rothenberger, A. (2004). Training of slow cortical potentials in attention-deficit/hyperactivity disorder: evidence for positive behavioral and neurophysiological effects. *Biological psychiatry* 55, 772–775. doi:10.1016/j.biopsych.2003.11.013

Holtmann, M., Grasmann, D., Cionek-Szpak, E., Hager, V., Panzner, N., Beyer, A., et al. (2009). Spezifische wirksamkeit von neurofeedback auf die impulsivität bei adhs. *Kindheit und Entwicklung* 18, 95–104. doi:10.1026/0942-5403.18.2.95

James, G., Witten, D., Hastie, T., and Tibshirani, R. (2013). An introduction to statistical learning (Springer), vol. 112, chap. 8. 303–332

Linden, M., Habib, T., and Radojevic, V. (1996). A controlled study of the effects of eeg biofeedback on cognition and behavior of children with attention deficit disorder and learning disabilities. *Applied Psychophysiology and Biofeedback* 21, 35–49

Maurizio, S., Liechti, M. D., Heinrich, H., Jäncke, L., Steinhausen, H.-C., Walitza, S., et al. (2014). Comparing tomographic eeg neurofeedback and emg biofeedback in children with attention-deficit/hyperactivity disorder. *Biological psychology* 95, 31–44. doi:10.1016/j.biopsycho.2013.10.008

Montgomery, D. C., Peck, E. A., and Vining, G. G. (2012). Introduction to linear regression analysis (John Wiley & Sons), vol. 821, chap. 5. 190–191

Morris, S. B. (2008). Estimating effect sizes from pretest-posttest-control group designs. *Organizational Research Methods* 11, 364–386. doi:10.1177/1094428106291059

Quinlan, J. R. (1986). Induction of decision trees. *Machine learning* 1, 81–106

Steiner, N. J., Frenette, E. C., Rene, K. M., Brennan, R. T., and Perrin, E. C. (2014). Neurofeedback and cognitive attention training for children with attention-deficit hyperactivity disorder in schools. *Journal of Developmental & Behavioral Pediatrics* 35, 18–27. doi:10.1097/DBP.0000000000000009

Steiner, N. J., Sheldrick, R. C., Gotthelf, D., and Perrin, E. C. (2011). Computer-based attention training in the schools for children with attention deficit/hyperactivity disorder: a preliminary trial. *Clinical pediatrics* 50, 615–622. doi:10.1177/0009922810397887

Strehl, U., Aggensteiner, P., Wachtlin, D., Brandeis, D., Albrecht, B., Arana, M., et al. (2017). Neurofeedback of slow cortical potentials in children with attention-deficit/hyperactivity disorder: A multicenter randomized trial controlling for unspecific effects. *Frontiers in human neuroscience* 11. doi:10.3389/fnhum.2017.00135

Tibshirani, R. (1996). Regression shrinkage and selection via the lasso. *Journal of the Royal Statistical Society. Series B (Methodological)* , 267–288

van Dongen-Boomsma, M., Vollebregt, M. A., Slaats-Willemse, D., and Buitelaar, J. K. (2013). A randomized placebo-controlled trial of electroencephalographic (eeg) neurofeedback in children with attention-deficit/hyperactivity disorder. *J Clin Psychiatry* 74, 821–827. doi:10.4088/JCP.12m08321
